# Coronary artery size based on intravascular ultrasound in Southeast Asia population

**DOI:** 10.1186/s43044-024-00543-w

**Published:** 2024-08-22

**Authors:** Aninka Saboe, Minsy Titi Sari, Achmad Fauzi Yahya, Muhammad Rizki Akbar

**Affiliations:** Department of Cardiology and Vascular Medicine, Universitas Padjadjaran - Dr Hasan Sadikin Hospital, Jalan Eyckman 38, Bandung, 40161 Indonesia

**Keywords:** Coronary artery size, Southeast Asia, Intravascular ultrasound, Predictor of coronary artery size

## Abstract

**Background:**

One of the downsides of percutaneous coronary intervention (PCI) is stent failure which could be related to stent underexpansion. Hence, PCI based on an accurate coronary artery size obtained from intracoronary imaging is tremendously important. Until now, there is no data about all coronary artery dimensions in the Southeast Asian population performed by intravascular ultrasound (IVUS). The coronary artery size of 153 patients with chronic coronary syndrome (CCS) in acute or chronic settings who underwent percutaneous coronary intervention (PCI) with IVUS was examined. The mean artery size and its predictors were analyzed.

**Results:**

There were 153 patients with 633 coronary artery segments: the mean left main (LM) external elastic membrane (EEM) diameter and cross-sectional area (CSA) were 5.02 ± 0.43 mm and 19.93 ± 3.48 mm^2^, proximal left anterior descending artery (LAD) 4.25 ± 0.42 mm and 14.34 ± 2.85 mm^2^, the mid-LAD 3.86 ± 0.39 mm and 11.70 ± 2.24 mm^2^, the distal LAD 3.32 (2.83–4.30) mm and 8.77(6.23–14.99) mm^2^, the proximal left circumflex artery (LCX) 3.91 ± 0.42 mm and 12.07 ± 2.53 mm^2^, the distal LCX 3.51 ± 0.47 mm and 9.90 (5.09–14.20) mm^2^, the proximal right coronary artery (RCA) 4.50 ± 0.48 mm and 16.14 ± 3.43 mm^2^, the mid-RCA 4.16 ± 0.420 mm and 13.74 ± 2.72 mm^2^, the distal RCA 3.81 ± 0.41 mm and 11.59 ± 2.46 mm^2^, respectively. Body surface area (BSA) is an independent predictor for the majority of epicardial coronary arteries with a positive linear relationship.

**Conclusions:**

The mean artery size of the Indonesian population was comparable with previous studies. The knowledge of coronary artery size will help the clinician to have a reference for intervention, especially when no intravascular imaging is available.

## Background

Percutaneous coronary intervention (PCI) is a recommended procedure for the symptomatic chronic coronary syndrome (CCS) patients after optimal medications and or to improve prognosis [1]. Angiography is one of the conventional modalities to evaluate coronary anatomy and to guide PCI procedures. But, despite being widely available, it has shortcomings which are based on a luminogram and two dimensions. Hence, it will underestimate the true vessel size and lead to improper stent sizing and expansion, increasing the risk of stent failure [2, 3].

Intravascular imaging provides more accurate images and measurements of artery dimensions than conventional angiography [3]. Intravascular imaging (IVUS) has a deeper tissue penetration compared to optical coherence tomography (OCT), hence a better view of vessel size [4]. PCI guided by intracoronary imaging has a lower target vessel failure (TVF) and major adverse cardiovascular event (MACE) compared to angiography [4, 5]. But until now, most of the procedures are still guided by angiography rather than intracoronary imaging [6, 7]. The main reasons are higher costs, longer time procedural, lack of training, risk of complication, and the need for contrast in OCT [8]. Optimal stent sizing using intracoronary imaging can be determined practically by measuring the distal reference lumen or external elastic membrane (EEM) diameter. Adequate stent expansion can be evaluated by comparing the minimal stent area (MSA) with the reference lumen or EEM area [2, 4].

On the other side, several factors influence the coronary artery size such as race, body surface area (BSA), gender, and environment [9–14]. Studies about vessel size showed variable results, some studies revealed that Asian and Caucasian populations have no significant difference after matching baseline characteristics between groups but another study showed that the Asian race has a smaller vessel size even after adjusting for BSA [10, 15–17]. Besides, there is a different target of MSA in the left main (LM) PCI between Asians and Caucasian populations [10, 11].

Until now, there is no study evaluating all epicardial coronary artery sizes using IVUS in the Southeast Asia population. The aims of this study were: (i) to investigate the coronary artery size (diameter and cross-sectional area/ CSA) at the segment free of significant atherosclerosis by IVUS according to American Heart Association (AHA) coronary artery segmentation in the Indonesian population, especially West Java using IVUS; (ii) to conduct a multivariate analysis of the demographic factors that could be a predictor for coronary artery size.

## Methods

### Study design

This retrospective cross-sectional study is performed in the Department of Cardiology and Vascular Medicine, Universitas Padjadjaran—Hasan Sadikin General Hospital, Bandung Indonesia. 238 CCS patients underwent IVUS-guided PCI. Inclusion criteria are segments free of significant atherosclerosis (< 50% plaque area). Left dominance system, coronary anomalies, incomplete image, unable to obtain ≥ 180° for diameter assessment, and ≥ 270° for CSA measurement were excluded.

This study was conducted according to the principles of the Declaration of Helsinki. All patients provided written informed consent and approved by the Medical Research Ethics Committee Dr. Hasan Sadikin General Hospital, West Java, Indonesia (LB.02.01/X.6.5/17/2022).

### Data collection

Demographic data was collected from medical records consisting of age, gender, body weight, body height, body mass index (BMI), BSA, cardiovascular risk factors, and left ventricular mass index (LVMI).

Patients were categorized as CCS if there was obstructive or non-obstructive atherosclerotic disease of epicardial arteries which came with symptoms of angina and/or dyspnea, history of acute coronary syndrome, recent revascularization, or left ventricular dysfunction [20]. Hypertension was established as systolic blood pressure ≥ 140 mmHg and or diastolic blood pressure ≥ 90 mmHg following 2–3 office visits or on anti-hypertensive medication [21]. Dyslipidemia was defined as total cholesterol ≥ 200 mg/dl, low-density lipoprotein C (LDL-C) ≥ 130 mg/dl, high-density lipoprotein (HDL) < 40 mg/dl for men and < 50 mg/dl for women, triglyceride > 150 mg/dl or the history of using anti-hyperlipidemia drugs [22]. Diabetes mellitus type 2 (DMT2) was defined as fasting blood sugar ≥ 126 mg/dl, random blood sugar ≥ 200 mg/dl, or HbA1c ≥ 6.5% or using an anti-hyperglycemia agent [23]. Smoker was categorized as currently or past smoking cigarettes.

### Gray-scale IVUS analysis

**Fig. 1:**
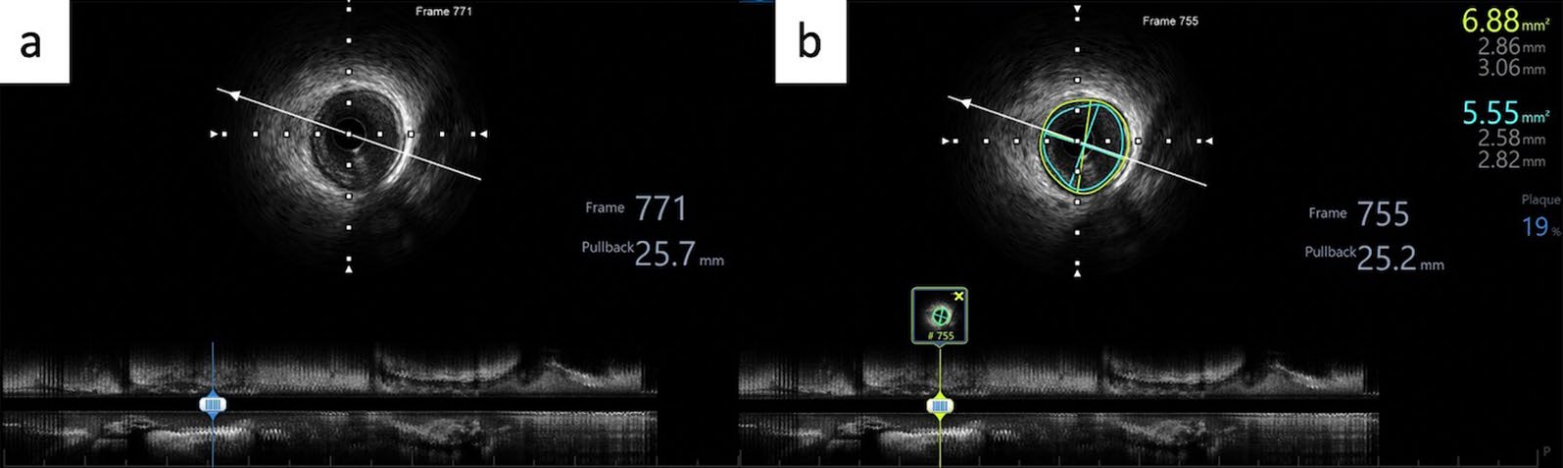
IVUS Image Acquisition and Measurement. **A** IVUS longitudinal view; **B** EEM CSA tracing and diameter measurement. CSA = cross-sectional area; EEM = external elastic membrane; IVUS = intravascular ultrasound

After intravenous nitroglycerine administration, IVUS was performed using OptiCross 40 mHz Coronary Imaging Catheter, Boston Scientific. The pullback was taken starting 15 mm distal to the lesion till the aorta-ostial junction using an automatic pullback at a speed of 0.5 mm/s. PCI was performed as per standard procedure. The IVUS dicom was analyzed by one independent observer who was not aware of patient details using a *post-processing Boston Scientific image viewer*. Each artery segment was determined with the guidance of the placement of an IVUS catheter from angiography. The external elastic membrane (EEM) cross-sectional area (CSA) and diameter were measured within 10–15 mm proximal before any side branch with plaque burden < 50% to avoid vessel remodeling. After an automatic border detection for EEM, a manual correction was done for CSA measurement, and the mean diameter of the longest and shortest diameter across the center point of the lumen through EEM was taken for diameter measurement (Fig. 1).

### Statistical analysis

All statistical analyses were performed using SPSS version 26.0 (IBM Corp, Armonk, NY, United States). Categorical data were presented as percentages (%) and frequencies. The distribution of numerical data was analyzed by the Kolmogrov-Smirnov test. For normal distribution, presented as mean ± standard deviation, if skewed distribution reported as median and interquartile range. An unpaired t-test was used to compare between groups for normal distribution and Mann Whitney U test for skewed distribution. The predictor for coronary artery size was analyzed using multi-linear regression, expressed in Pearson beta coefficient. A p-value < 0.05 was considered statistically significant.

## Results

**Fig. 2:**
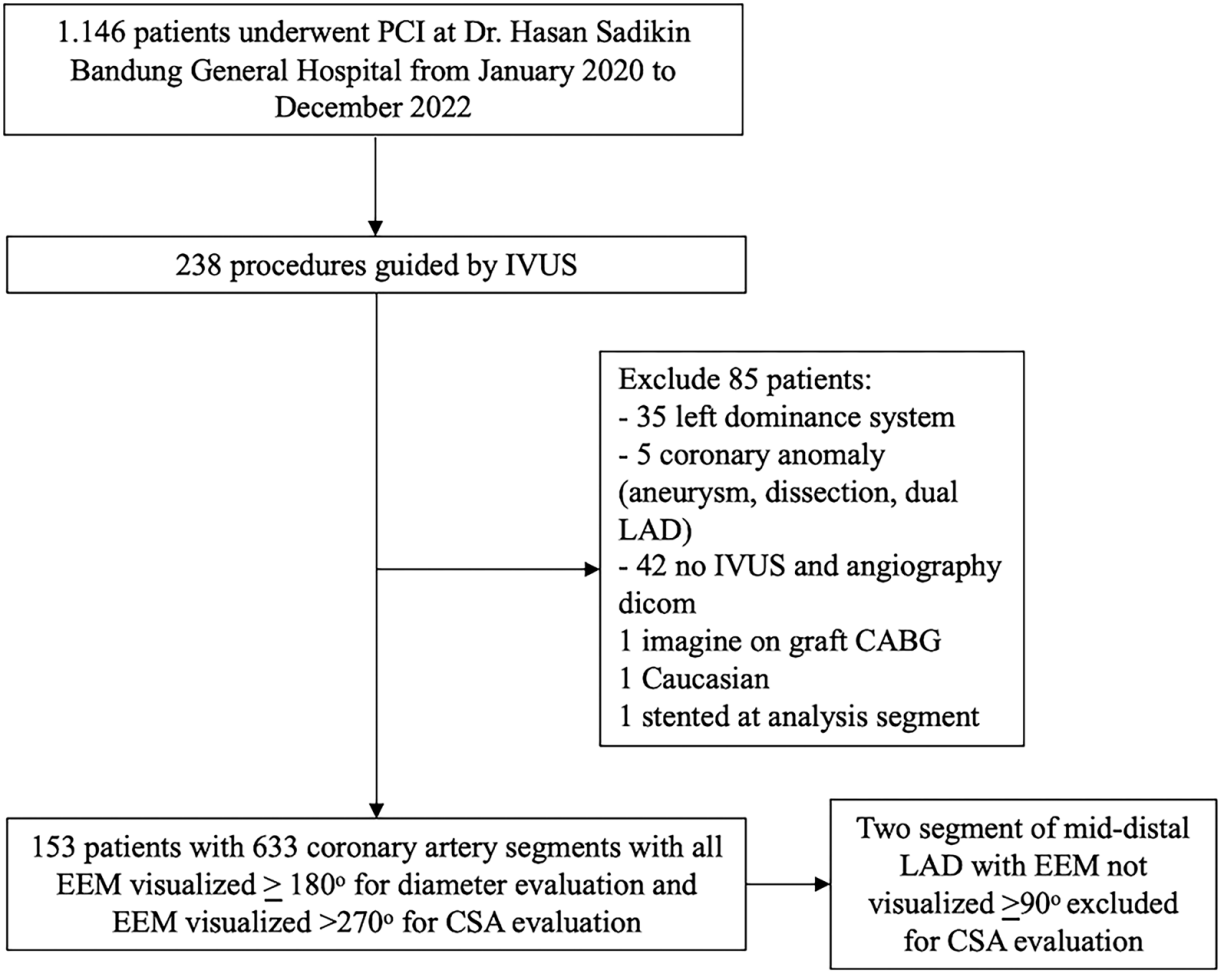
Patient enrollment. CABG = coronary artery bypass graft; CSA = cross sectional area; EEM = external elastic membrane; IVUS = intravascular ultrasound; LAD = left anterior descending; PCI = percutaneous coronary intervention

From a total of 1.146 patients who underwent PCI at Dr. Hasan Sadikin General Hospital, 20.8% (238 patients) were guided by IVUS. We excluded 35 patients with a left dominance system, 5 coronary anomalies, 42 incomplete IVUS/angiographic data, 1 image on a grafted vessel, 1 Caucasian, and 1 stented at the analysis segment. The final sample was 153 patients with 633 coronary artery segments. Figure 2 shows the schematic of patient enrollment.

The mean age of the patients was 59.92 ± 10.29 years old. Seventy-five percent were males. The mean BSA was 1.71 ± 0.18 m^2^ with a BMI of 24.67 ± 4.02 kg/m^2^. The majority had hypertension (64.1%), followed by smoking (62.7%), dyslipidemia (36.6%), and DMT2 20.9%. Most of the patients had LVMI 134.74 ± 48.35 g/m^2^, categorized as hypertrophy. The baseline characteristics are shown in Table 1.

**Table 1:** Baseline Characteristics of the Patients

Variable	N = 153
Age (year)	59.92 ± 10.29
Sex	
Males (n, %)	116(75.8%)
Females (n, %)	37(24.2%)
Body weight (kg)	65(34.00–101.00)
Body height (cm)	162.39 ± 7.86
BSA (m^2^)	1.71 ± 0.18
BMI (kg/m^2^)	24.67 ± 4.02
Hypertension (n, %)	98(64.1%)
DMT2 (n, %)	32(20.9%)
Smoking (n, %)	96(62.7%)
Dyslipidemia (n, %)	56(36.6%)
LVMI (n = 168) (gr/m^2^)	134.74 ± 48.35

Data presented as number (%), mean ± standard deviation, or median (interquartile range);

BMI = body mass index; BSA = body surface area; DMT2 = diabetes mellitus type 2; LVMI = left ventricular mass index

From the total of 633 coronary segments analyzed, the most frequent IVUS usage was at the Left Main (LM) which was 100 segments, followed by proximal Left Anterior Descending (LAD) 93 segments, mid-LAD 89 segments, distal LAD 85 segments, proximal Left Circumflex (LCX) 39 segments, distal LCX 35 segments, proximal Right Coronary Artery (RCA) 66 segments, mid-RCA 65 segments, distal RCA 61 segments.

The mean LM EEM diameter and CSA were 5.02 ± 0.43 mm and 19.93 ± 3.48 mm^2^, proximal LAD 4.25 ± 0.42 mm and 14.34 ± 2.85 mm^2^, the mid-LAD 3.86 ± 0.39 mm and 11.70 ± 2.24 mm^2^, the distal LAD 3.32 (2.83–4.30) mm and 8.77(6.23–14.99) mm^2^, the proximal LCX 3.91 ± 0.42 mm and 12.07 ± 2.53 mm^2^, the distal LCX 3.51 ± 0.47 mm and 9.90 (5.09–14.20) mm^2^, the proximal RCA 4.50 ± 0.48 mm and 16.14 ± 3.43 mm^2^, the mid-RCA 4.16 ± 0.42 mm and 13.74 ± 2.72 mm^2^, the distal RCA 3.81 ± 0.41 mm and 11.59 ± 2.46 mm^2^, respectively. The largest vessel was LM, followed by proximal RCA, proximal LAD, mid-RCA, proximal LCX, mid-LAD, distal RCA, distal LCX, and distal LAD (Table 2).

**Table 2:** Mean EEM Coronary Artery

Artery	LM (100)	Proximal LAD (93)	Mid-LAD (89)	Distal LAD (85)	Proximal LCX (39)
Diameter (mm)	5.02 ± 0.43	4.25 ± 0.42	3.86 ± 0.39	3.32 (2.83–4.30)	3.91 ± 0.42
CSA (mm^2^)	19.93 ± 3.48	14.34 ± 2.85	11.70 ± 2.24	8.77 (6.23–14.99)	12.07 ± 2.53

Artery	Distal LCX (35)	Proximal RCA (66)	Mid-RCA (65)	Distal RCA (61)
Diameter (mm)	3.51 ± 0.47	4.50 ± 0.48	4.16 ± 0.42	3.81 ± 0.41
CSA (mm^2^)	9.90 (5.09–14.20)	16.14 ± 3.43	13.74 ± 2.72	11.59 ± 2.46

Data presented as mean ± standard deviation, or median (interquartile range); CSA = cross-sectional area; EEM = external elastic membrane LAD = left anterior descending; LCX = left circumflex; LM = left main; RCA = right coronary artery

Tables 3 and 4 showed the comparison of baseline demographics with mean artery size (EEM diameter and CSA). Generally, males had a greater artery size compared to females with mid, distal LAD, proximal, and distal LCX reaching a statistically significant difference (p-value < 0.05). Diabetes, hypertension, and smoking did not influence the artery size in this study. On the other hand, dyslipidemia patients had smaller artery sizes, especially the LCX.

**Table 3:** Comparison of EEM Diameter with Baseline Demographic by IVUS

	EEM Diameter LM (mm)	p-value	EEM Diameter Proximal LAD (mm)	p-value	EEM Diameter Mid LAD (mm)	p-value	EEM Diameter Distal LAD (mm)	p-value
Male	5.07 ± 0.403	0.077	4.29 ± 0.388	0.105	3.91 ± 0.368	0.020*	3.43(2.86–4.30)	0.010*
Female	4.89 ± 0.496		4.13 ± 0.512		3.70 ± 0.413		3.19(2.83–3.83)	
Diabetes	5.01 ± 0.506	0.869	4.29 ± 0.440	0.623	3.85 ± 0.441	0.909	3.33(2.89–4.30)	0.888
Non-diabetes	5.03 ± 0.413		4.24 ± 0.427		3.86 ± 0.374		3.32(2.83–4.12)	
Dyslipidemia	4.83 (3.93–5.86)	0.110	4.22 ± 0.491	0.643	3.83 ± 0.429	0.633	3.23(2.84–4.30)	0.887
Non- dyslipidemia	5.11(4.00–6.28)		4.27 ± 0.392		3.87 ± 0.367		3.39(2.83–3.96)	
Hypertensive	5.09 ± 0.408	0.059	4.28 ± 0.430	0.315	3.86 ± 0.401	0.808	3.38(2.83–4.30)	0.220
Non-hypertensive	4.92 ± 0.453		4.19 ± 0.425		3.84 ± 0.372		3.22(2.86–4.02)	
Smoking	5.03 ± 0.402	0.971	4.22 ± 0.387	0.345	3.87 ± 0.381	0.716	3.38(2.86–3.93)	0.536
Non-smoking	5.03 ± 0.483		4.30 ± 0.487		3.84 ± 0.406		3.21(2.83–4.30)	

	EEM Diameter Proximal LCX (mm)	p-value	EEM Diameter Distal LCX (mm)	p-value	EEM Diameter Proximal RCA (mm)	p-value	EEM Diameter Mid RCA (mm)	p-value	EEM Diameter Distal RCA (mm)	p-value
Male	3.98 ± 0.400	0.032*	3.60 ± 0.423	0.022*	4.53 ± 0.453	0.305	4.18 ± 0.408	0.357	3.84 ± 0.384	0.262
Female	3.63 ± 0.384		3.15 ± 0.530		4.37 ± 0.585		4.05 ± 0.481		3.69 ± 0.506	
Diabetes	3.74 ± 0.330	0.273	3.35 ± 0.457	0.407	4.57 ± 0.435	0.566	4.28 ± 0.377	0.271	3.87 ± 0.366	0.587
Non-diabetes	3.94 ± 0.428		3.54 ± 0.480		4.48 ± 0.490		4.13 ± 0.427		3.80 ± 0.419	
Dyslipidemia	3.70 ± 0.404	0.034*	3.27 ± 0.396	0.039*	4.39 ± 0.452	0.167	4.15(2.98–4.67)	0.501	3.70 ± 0.378	0.094
Non- dyslipidemia	4.00 ± 0.395		3.63 ± 0.474		4.56 ± 0.489		4.20 ± 0.448		3.88 ± 0.417	
Hypertensive	3.93 ± 0.424	0.690	3.52 ± 0.501	0.914	4.47 ± 0.537	0.585	4.12 ± 0.459	0.369	3.78 ± 0.435	0.517
Non-hypertensive	3.87 ± 0.417		3.50 ± 0.441		4.54 ± 0.385		4.22 ± 0.354		3.85 ± 0.372	
Smoking	4.01 ± 0.352	0.072	3.61 ± 0.393	0.154	4.54 ± 0.464	0.295	4.18 ± 0.424	0.506	3.84 ± 0.414	0.449
Non-smoking	3.76 ± 0.478		3.35 ± 0.567		4.41 ± 0.506		4.11 ± 0.417		3.76 ± 0.403	

Data presented as mean ± standard deviation or median (interquartile range); *p-value < 0.05

EEM = external elastic membrane; IVUS = intravascular ultrasound; LAD = left anterior descending; LCX = left circumflex; LM = left main; RCA = right coronary artery

**Table 4:** Comparison of EEM CSA with Baseline Demographic by IVUS

	EEM CSA LM (mm2)	p-value	EEM CSA Proximal LAD (mm2)	p-value	EEM CSA Mid LAD (mm2)	p-value	EEM CSA Distal LAD (mm2)	p-value
Male	20.27 ± 3.352	0.086	14.64 ± 2.550	0.053	12.04 ± 2.150	0.013*	9.27(6.61–14.99)	0.011*
Female	18.84 ± 3.758		13.09(9.43–24.55)		10.71 ± 2.230		8.28 ± 1.553	
Diabetes	19.82 ± 3.950	0.862	14.72 ± 2.930	0.494	11.79 ± 2.477	0.832	8.98(6.73–14.99)	0.653
Non-diabetes	19.97 ± 3.382		14.23 ± 2.847		11.67 ± 2.176		8.64(6.23–13.20)	
Dyslipidemia	19.12 ± 3.771	0.079	14.19 ± 3.327	0.693	11.58 ± 2.537	0.704	8.26(6.27–14.99)	0.993
Non- dyslipidemia	20.40 ± 3.250		14.43 ± 2.568		11.77 ± 2.058		9.02 ± 1.579	
Hypertensive	20.36 ± 3.358	0.107	14.57 ± 2.785	0.269	14.57 ± 2.785	0.704	9.24 ± 1.788	0.143
Non-hypertensive	19.19 ± 3.619		13.87 ± 2.806		13.87 ± 2.806		8.11(6.61–12.71)	
Smoking	19.95 ± 3.382	0.960	14.14 ± 2.515	0.431	11.72 ± 2.063	0.883	9.05 ± 1.390	0.557
Non-smoking	19.91 ± 3.702		14.67 ± 3.333		11.65 ± 2.508		8.09(6.23–14.99)	

	EEM CSA Proximal LCX (mm2)	p-value	EEM CSA Distal LCX (mm2)	p-value	EEM CSA Proximal RCA (mm2)	p-value	EEM CSA Mid RCA (mm2)	p-value	EEM CSA Distal RCA (mm2)	p-value
Male	12.50 ± 2.516	0.034*	10.35 ± 2.421	0.039*	9.13 ± 3.177	0.290	13.89 ± 2.701	0.339	11.75 ± 2.349	0.271
Female	10.40 ± 1.901		8.00 ± 2.947		10.33 ± 2.274		13.02 ± 2.844		10.84 ± 2.918	
Diabetes	11.13 ± 1.698	0.324	8.95 ± 2.478	0.404	16.57 ± 3.042	0.632	14.51 ± 2.554	0.283	12.12 ± 2.190	0.434
Non-diabetes	12.24 ± 2.637		10.04 ± 2.702		16.04 ± 3.528		13.57 ± 2.752		11.47 ± 2.520	
Dyslipidemia	10.89 ± 2.341	0.051	8.42 ± 2.048	0.026*	15.34 ± 2.977	0.141	13.77(7.16–16.70)	0.497	10.99 ± 2.252	0.127
Non- dyslipidemia	12.60 ± 2.472		10.55 ± 2.681		16.62 ± 3.626		14.04 ± 2.995		11.98 ± 2.539	
Hypertensive	12.18 ± 2.589	0.703	9.97 ± 2.826	0.791	15.93 ± 3.841	0.568	13.51 ± 2.921	0.413	11.36 ± 2.559	0.401
Non-hypertensive	11.85 ± 2.498		9.71 ± 2.435		16.43 ± 2.770		14.08 ± 2.408		11.91 ± 2.321	
Smoking	12.62 ± 2.202	0.087	10.33 ± 2.274	0.169	16.50 ± 3.427	0.231	13.94 ± 2.796	0.405	11.81 ± 2.499	0.331
Non-smoking	11.19 ± 2.842		7.77(5.09–13.72)		15.42 ± 3.395		13.34 ± 2.589		11.16 ± 2.382	

Data presented as mean ± standard deviation or median (interquartile range); *p-value < 0.05

CSA = cross-sectional area; EEM = external elastic membrane; IVUS = intravascular ultrasound; LAD = left anterior descending; LCX = left circumflex; LM = left main; RCA = right coronary artery

After multiple linear regression analyses, BSA was an independent predictor for most of the artery sizes except LCX with a positive linear relationship. Age was a positive independent predictor for EEM diameter (β 0.008, 95% CI 0.001–0.014 and p 0.017) and CSA of distal LAD (β 0.041, 95% CI 0.008–0.074 and p 0.017). The gender variable shows female was a negative independent predictor for EEM diameter of distal LCX (β − 0.453, 95% CI − 0.835–(− 0.071 and p 0.022) and CSA of proximal LCX (β − 2.101, 95% CI − 4.039–(− 1.163) and p 0.034). Hypertension was a positive independent predictor for the EEM diameter of LM (β 0.172, 95% CI 0.006–0.337 and p 0.043). Dyslipidemia was a negative independent predictor for the EEM diameter of proximal LCX (β − 0.292, 95% CI − 0.563–(− 0.021) and p 0.036) and CSA of distal LCX (β − 2.128, 95% CI − 3.984–(− 0.272) and p 0.026). The significant variables that predict the vessel size are shown in Tables 5, 6, 7 and 8.

**Table 5:** P-value of Variables Predictor for EEM Diameter (mm) with Backward Selection

Artery	P-value								
	LM	Proximal LAD	Mid LAD	Distal LAD	Proximal LCX	Distal LCX	Proximal RCA	Mid RCA	Distal RCA
Age	0.756	0.565	0.290	0.017*	0.511	0.768	0.534	0.508	0.420
Gender	0.955	0.302	0.255	0.125	0.504	0.022*	0.505	0.565	0.939
BMI	0.890	0.589	0.811	0.706	0.495	0.560	0.651	0.129	0.977
BSA	0.002*	0.004*	0.011*	0.001*	0.739	0.689	0.004*	0.003*	0.019*
DMT2	0.380	0.736	0.873	0.995	0.252	0.412	0.954	0.463	0.734
HT	0.043*	0.295	0.980	0.551	0.352	0.916	0.655	0.466	0.690
Dyslipidemia	0.473	0.561	0.706	0.784	0.036*	0.158	0.33	0.515	0.155
Smoking	0.791	0.239	0.834	0.448	0.253	0.459	0.753	0.618	0.927

BMI = body mass index; BSA = body surface area; EEM = external elastic membrane; DMT2 = diabetes mellitus type 2; HT = hypertension; IVUS = intravascular ultrasound; LAD = left anterior descending; LCX = left circumflex; LM = left main; RCA = right coronary artery; *p-value < 0.05

**Table 6:** P-value of Variables Predictor for EEM CSA (mm^2^) with Backward Selection

Artery	P-value								
	LM	Proximal LAD	Mid LAD	Distal LAD	Proximal LCX	Distal LCX	Proximal RCA	Mid RCA	Distal RCA
Age	0.567	0.596	0.163	0.017*	0.423	0.736	0.781	0.681	0.627
Gender	0.929	0.473	0.177	0.104	0.034*	0.252	0.338	0.559	0.875
BMI	0.885	0.580	0.888	0.716	0.577	0.551	0.528	0.136	0.847
BSA	0.000*	0.004*	0.011*	0.001*	0.900	0.746	0.002*	0.005*	0.017*
DMT2	0.449	0.583	0.874	0.686	0.344	0.395	0.852	0.446	0.588
HT	0,071	0.321	0.935	0.498	0.358	0.790	0.730	0.589	0.646
Dyslipidemia	0.378	0.606	0.700	0.668	0.075	0.026*	0.266	0.438	0.199
Smoking	0.825	0.182	0.618	0.293	0.280	0.669	0.678	0.750	0.932

BMI = body mass index; BSA = body surface area; CSA = cross-sectional area; EEM = external elastic membrane; DMT2 = diabetes mellitus type 2; HT = hypertension; IVUS = intravascular ultrasound; LAD = left anterior descending; LCX = left circumflex; LM = left main; RCA = right coronary artery; *p-value < 0.05

**Table 7:** Multiple regression analysis of variable(s) associated with EEM diameter (mm) based on linear regression with backward selection method

Vessel	β	SE	95% CI for β	p-Value
LM model	2.878 (constanta)			
BSA	1.293	0.401	0.496–2.089	0.002*
HT	0.172	0.083	0.006–0.337	0.043*
Prox LAD model	3.014 (constanta)			
BSA	0.726	0.248	0.234–1.219	0.004*
Mid LAD model	2.809 (constanta)			
BSA	0.617	0.238	0.145–1.090	0.011*
Distal LAD model	1.687 (constanta)			
BSA	0.729	0.204	0.323–1.135	0.001*
Age	0.008	0.003	0.001–0.014	0.017*
Prox LCX model	3.857 (constanta)			
Dyslipidemia	− 0.292	0.134	− 0.563–(− 0.021)	0.036*
Distal LCX model	3.605 (constanta)			
Gender^#^	− 0.453	0.188	− 0.835–(− 0.071)	0.022*
Prox RCA model	3.018 (constanta)			
BSA	0.866	0.291	0.285–1.448	0.004*
Mid RCA model	2.781 (constanta)			
BSA	0.804	0.258	0.288–1.320	0.003*
Distal RCA model	2.795 (constanta)			
BSA	0.635	0.264	0.107–1.163	0.019*

^#^: Gender code = 0 for males and 1 for females; *Only the significant variable with p-value < 0.05 was shown

BSA = body surface area; CI = confidence interval; EEM = external elastic membrane; LAD = left anterior descending; LCX = left circumflex; LM = left main; RCA = right coronary artery; SE = standard of error

**Table 8:** Multiple Regression Analysis of Variable(s) Associated with EEM CSA (mm^2^) Based on Linear Regression with Backward Selection Method

Vessel	β	SE	95% CI for β	p-Value
LM model	2.745 (constanta)			
BSA	9.601	1.826	5.977–13.225	0.000*
Prox LAD model	6.124 (constanta)			
BSA	4.833	1.653	1.548–8.117	0.004*
Mid LAD model	5.659 (constanta)			
BSA	3.560	1.365	0.847–6.273	0.011*
Distal LAD model	0.011 (constanta)			
Age	0.041	0.017	0.008–0.074	0.017*
BSA	3.912	1.104	1.716–6.108	0.001*
Prox LCX model	12.502 (constanta)			
Gender#	− 2.101	0.957	− 4.039–(− 1.163)	0.034*
Distal LCX model	10.552 (constanta)			
Dyslipidemia	− 2.128	0.912	− 3.984–(− 0.272)	0.026*
Prox RCA model	4.864 (constanta)			
BSA	6.600	2.065	2.475–10.725	0.002*
Mid RCA model	5.235 (constanta)			
BSA	4.965	1.687	1.595–8.335	0.005*
Distal RCA model	4.810 (constanta)			
BSA	3.960	1.610	0.739–7.181	0.017*

^#^Gender code = 0 for males and 1 for females; *Only the significant variable with p-value < 0.05 was shown

BSA = body surface area; CI = confidence interval; CSA = cross-sectional area; EEM = external elastic membrane; LAD = left anterior descending; LCX = left circumflex; LM = left main; RCA = right coronary artery; SE = standard of error

## Discussion

Coronary artery size is generally influenced by many factors including genetics, age, gender, body surface area, LVMI, and environment [9, 11, 15–17, 24–26]. Several reports found that the artery size of the Asian population was smaller than Caucasians, but mostly due to smaller BSA [15–17, 27]. Table 9 shows the artery size of various studies comparing the Asian and White populations. The artery dimensions of Asians were similar to the White race. The mean EEM CSA and diameter of LM and proximal epicardial artery in our study were smaller than most of the Indian population but comparable to the Asian populations in the study of Rusinova, et al. On the other side, a study from Kim et al. found a smaller LM size than in our study. It could be caused by different inclusion criteria, such as the Reddy et al. including coronary artery segment with < 20% atheroma, the study from Punamiya et al. including plaque burden ± 30%, study from Reni P et al. including diseased LM with total plaque burden 40–70% [11, 17, 26]. In our study, we include the coronary artery segment with < 50% plaque burden. Besides, the larger the BSA, the larger the vessel size.

**Table 9:** Comparison of artery size (external elastic membrane) by IVUS

Authors	Population	Number sample	BSA (m^2^)	LM	Proximal LAD	Proximal LCX	Proximal RCA
Reddy et al. [11]	Indian	303	1.76 ± 0.17	n = 221 CSA: 25.11 ± 6.43 mm^2^	n = 164 CSA: 16.98 ± 4.87 mm^2^	n = 45 CSA: 15.56 ± 5.07 mm^2^	n = 62 CSA: 18.44 ± 4.79 mm^2^
Punamiya et al. [26]	Indian	140	-	n = 140 D: 5.53 ± 0.63 mm CSA: 24.79 ± 5.5 mm^2^	-	–	–
Rusinova RP et al. [17]	White and Asian	198	Asia: 1.7 ± 0.1 Kaukasia: 1.9 ± 0.2	n = 99 CSA Asian: 20.7 ± 4.5 mm^2^ n = 99 CSA White: 19.3 ± 4.2 mm^2^	-	–	–
Kim SG et al. [9]	White	257	1.94 ± 0.20	n = 257 CSA: 18.89 ± 4.44 mm^2^	-	–	–
Goel PK et al. [3]	Indian	186	-	n = 186 D: 4.33 ± 0.32 mm	n = 177 D: 3.61 ± 0.21 mm	n = 44 D: 3.31 ± 0.16 mm	-
Sheifer SE et al. [28]	White	75	1.92 ± 0.17	n = 75 CSA: 24.24 ± 6.29 mm^2^	n = 75 CSA: 17.31 ± 5.35 mm^2^	-	-
Saboe et al. (current study)	Indonesian	153	1.71 ± 0.18	n = 100 D: 5.02 ± 0.43 mm CSA: 19.93 ± 3.48 mm^2^	n = 93 D: 4.25 ± 0.42 mm CSA: 14.34 ± 2.85 mm^2^	n = 39 D: 3.91 ± 0.42 mm CSA: 12.07 ± 2.53 mm^2^	n = 66 D: 4.50 ± 0.48 mm CSA: 16.14 ± 3.43 mm^2^

CSA = Cross Section Area; D = Diameter; LAD = left anterior descending; LCX = left circumflex; LM = left main; RCA = right coronary artery

BSA consistently showed a positive correlation with the coronary dimension in many studies [9, 11, 16, 26]. The study from Punamiya found a linear relationship between BSA and LM coronary artery size. This present study also showed a positive independent predictor of BSA in the majority of epicardial coronary arteries, especially LM, LAD, and RCA. The insignificant of the LCX segment may be due to a smaller sample size. We developed the algorithm to explain the linear relationship of BSA toward artery size although they need more samples for testing and validation before clinical application.

Sheifer et al. analyzed the LM and LAD arteries of 75 patients using IVUS including the plaque burden ≤ 0.5, and found that after correction for BSA, females had smaller coronary dimensions than males. They suggest that there may be different estrogen levels that control the vascular tone and size [28]. Kim et al. identified from a total of 257 patients with < 20% atheroma, the LM artery in females was smaller than in males, even after adjusting for BSA, although BSA had a greater influence than sex [9]. This controversy to the study from Kornowski et al. found that in 718 patients after correcting for BSA, there were no differences of gender in coronary artery size and plaque burden [29]. The study from Reddy et al. also found no significant difference in artery size between genders after adjusting for BSA [11]. In our study, the males had a greater artery dimension than females with EEM CSA of proximal LCX and EEM diameter of distal LCX reaching a significant p-value < 0.05.

Hypertension is a significant predictor for LM EEM diameter (p-value 0.034). A study from Reddy et al. also found hypertension as a significant predictor for EEM CSA of LAD with a positive relationship. This is consistent with the study from Kozakova et al., which compared the LM CSA of 104 patients with hypertensive and physiologic LVH (athlete) from transesophageal echocardiography and found that in patients with hypertensive LVH, the LM CSA increased accordance to LV mass (r = 0.40, p < 0.01) and negatively correlated with the systolic blood pressure (r = − 0.48, p < 0.01). The physiologic concentric LVH will increase the coronary flow reserve (CFR), and so will the LM luminal diameter. However, in uncontrolled hypertension, the CFR was reduced due to artery remodeling and impaired endothelial function [12]. In this study, 64% of the patients had controlled blood pressure with a mean LVMI of 134.74 ± 48.35 gr/m^2^, with 48% categorized as concentric LVH. This is why the hypertensive patients had a greater LM EEM diameter.

The studies about coronary dimension and age yielded inconsistent results, with mostly came out with no correlation between them [13, 28, 30, 31]. Reddy et al. found that age was an independent predictor for LM and proximal LAD [11]. In our study, age was a significant independent predictor for distal LAD size with a weak positive association. Data from 98 necropsy patients found that age had a small predictor for EEM CSA which was the older patients had larger coronary arteries. This may be due to “senile dilatation” of coronary transverse and longitudinal dimensions [31].

The study from Paul et al. and Yasmin et al. found that the patients with higher BMI had a higher BMI had a smaller coronary artery size [13, 30]. The possible explanation is the higher the BMI, there is more adipose tissue deposits in the heart and a smaller coronary artery size [30]. In our study, BMI is not a significant predictor for coronary artery size, but patients with dyslipidemia had a smaller coronary dimension, especially for the EEM diameter of proximal LCX and CSA of distal LCX, which may be due to positive remodeling. More sample is needed to establish this relationship.

Neither diabetes nor smoking are independently correlated with artery size. The study from Punamiya et al. also did not find any significant predictor among those atherosclerosis risk factors [26].

This is the first study about all epicardial coronary arteries using IVUS. There was a study about all epicardial coronary arteries that compared their size between Caucasians and Asians, but it was conducted using coronary angiography instead of Intracoronary Imaging [15]. The artery sizes were smaller compared to our study because angiography measures the lumen of the artery instead of the EEM. Besides, the angiography is a dimension study and does not represent the true dimension of the artery. Hence, the accuracy of artery size estimation was less accurate than in our study.

## Conclusions

The mean artery sizes of the Indonesian population were comparable with previous studies of coronary artery size of Asian and White populations using IVUS. The BSA is an independent predictor for the majority of epicardial coronary arteries with a positive linear relationship. Males have a greater coronary artery dimension than females, which may be due to larger BSA. Hypertensive patients have a greater EEM diameter of LM with a positive linear relationship. Age is a predictor for distal LAD with a positive relationship. Dyslipidemia patients have a smaller vessel size and are an independent predictor for LCX. Neither diabetes nor smoking influences coronary artery size. Finally, the knowledge of coronary artery size will help the clinician to have a reference dimension for intervention, choosing the adequate device and stent size, especially when there is no intravascular imaging available.

### Strength

This is the first study of all epicardial coronary artery dimensions using IVUS.

### Limitation

This is a single-center observational study. The small sample precludes the ability to assess the accurate relationship of each variable on artery size. Only one independent observer was used to evaluate the artery size.

## Data Availability

The datasets used and/or analyzed during the current study are available from the corresponding author upon reasonable request.

## Abbreviations

AHA: American heart association
BMI: Body mass index
BSA: Body surface area
CABG: Coronary artery bypass graft
CCS: Chronic coronary syndrome
CI: Confidence interval
CFR: Coronary flow reserve
CSA: Cross sectional area
DMT2: Diabetes mellitus type 2
EEM: External elastic membrane
HDL: High-density lipoprotein
IVUS: Intravascular ultrasound
LAD: Left anterior descending
LCX: Left circumflex
LDL-C: Low-density lipoprotein-C
LM: Left main
LVMI: Left ventricular mass index
MACE: Major adverse cardiovascular event
MSA: Ainimal stent area
OCT: Optical coherence tomography
PCI: Percutaneous coronary intervention
RCA: Right coronary artery
SE: Standard of error
TVF: Target vessel failure
